# Secondhand smoke exposure and sleep disturbances among Korean adolescents: A nationally representative cross-sectional study

**DOI:** 10.18332/tid/213717

**Published:** 2025-12-31

**Authors:** Fengrui Hua, Yunyun Wu, Shengyuan Xu, Wenbin Du, Yunyun Xu

**Affiliations:** 1 Research Institute of Social Development, Southwestern University of Finance and Economics, Chengdu, China; 2 Graduate School of Information Sciences, Tohoku University, Sendai, Japan; 3 School of Social and Behavioral Sciences, Nanjing University, Nanjing, China

**Keywords:** secondhand smoke exposure, sleep disturbances, adolescents, South Korea

## Abstract

**INTRODUCTION:**

Inadequate sleep duration among adolescents is increasingly recognized as a significant public health concern, with potential associations with various environmental exposures. This study investigates the association between secondhand smoke (SHS) exposure and sleep duration in Korean adolescents.

**METHODS:**

This study employed a pooled cross-sectional design using data from the nationally representative Korea Youth Risk Behavior Web-based Survey from 2021 to 2024 (n=195664). This study analyzed self-reported sleep duration (hours/minutes) from the Korea Youth Risk Behavior Web-based Survey (KYRBS). Ordinary least squares regression models were used to analyze the association between SHS exposure days and sleep duration, adjusting for individual, school, and family-level control variables. The moderating effect of tobacco use was examined through interaction terms.

**RESULTS:**

SHS exposure demonstrated a significant negative association with adolescent sleep duration. After full adjustment for control variables, each additional day of SHS exposure was associated with a reduction in weekly average sleep duration (β= -0.027; 95% CI: -0.029 – -0.024, p<0.01), weekday sleep duration (β= -0.030; 95% CI: -0.03 – -0.028, p<0.01), and weekend sleep duration (β= -0.018; 95% CI: -0.023 – -0.013, p<0.01). Tobacco use significantly moderated this relationship, with the interaction term showing positive coefficients across all sleep duration models.

**CONCLUSIONS:**

SHS exposure significantly associated with sleep deprivation among Korean adolescents, with this association being influenced by individual tobacco use habits.

## INTRODUCTION

Adolescence represents a significant phase in an individual’s physical and mental maturation, with sufficient sleep playing a crucial role in cognitive function, emotional well-being, academic achievement, and long-term physical health^1,2^. The World Health Organization and national health authorities stress that adolescents require 9–10 hours of sleep per day^3^. Despite this recommendation, there is a growing global concern regarding sleep insufficiency among teenagers. For instance, average sleep duration is around 8 hours in Europe^4^, 7.3 hours in the United States^5^, 6.3 hours in Japan^6^, and 5–6 hours in Korea^7^. Secondhand smoke (SHS), also referred to as passive or involuntary smoking, entails non-smokers inhaling smoke either exhaled by smokers or from burning tobacco against their volition^8^. SHS exposure poses a significant health challenge during adolescence^9,10^. While numerous studies have established the adverse impact of active smoking on sleep^11,12^, there remains a dearth of research on the independent influence of SHS exposure on sleep patterns in adolescents, necessitating further exploration of the underlying mechanisms.

Adolescents commonly experience sleep disorders due to various factors such as circadian changes, academic stress, and electronic device use^12^. Environmental influences like SHS exposure play a significant role in this context. SHS comprises numerous chemicals, many of which possess neurotoxic or irritant properties^13^. Exposure to SHS can disrupt sleep patterns through physiological mechanisms like inducing or worsening respiratory inflammation and reducing blood oxygen saturation, ultimately impacting sleep duration and quality^14^. Unlike active smoking, passive and widespread exposure to SHS may have subtle effects on sleep that are easily underestimated by individuals and families^10,15^.

Despite the implementation of a smoke-free policy in Korea through the WHO Framework Convention on Tobacco Control in 2005, many Korean teenagers are still exposed to secondhand smoke in various settings such as homes, public places, and around schools. Data from the 16th Korean Adolescent Health Behavior Survey conducted in 2020 revealed that the rate of secondhand smoke exposure in adolescent families was 20.0% for boys and 22.4% for girls, surpassing rates observed in other countries^16,17^.

Adolescents who smoke often experience sleep disorders such as insufficient sleep duration and reduced sleep quality due to nicotine’s direct interference with sleep cycles^18,19^. Additionally, exposure to SHS negatively affects sleep health in non-smoking adolescents, potentially impairing sleep through environmental factors like respiratory irritation^13,20^. However, previous studies have primarily treated smoking and SHS exposure as separate issues, overlooking their potential co-occurrence and interactions at the individual level. In reality, among adolescents, SHS exposure frequently is associated with active smoking, and disregarding this relationship may lead to misinterpretations of secondhand smoke’s true health impacts^21,22^. Thus, an unexplored question is whether adolescents’ tobacco use behavior influences how SHS exposure affects their sleep health. This study introduces ‘tobacco use’ as a key variable to investigate the interplay between SHS exposure and adolescent sleep duration.

Therefore, this study aimed to utilize a large representative sample to comprehensively characterize the current status of SHS exposure and sleep patterns among Korean adolescents, evaluate the relationship between these factors, and specifically examine how adolescent tobacco use moderates this association.

## METHODS

### Data sources and sample selection

This cross-sectional study utilized pooled data from the 2021–2024 Korea Youth Risk Behavior Web-based Survey (KYRBS) to examine the association between SHS exposure and sleep duration in Korean adolescents. This study conducted a secondary analysis of data from the KYRBS, an anonymous, self-administered online survey implemented annually since 2005 by the Korea Disease Control and Prevention Agency (KDCA). The KYRBS applies a two-stage stratified cluster sampling design to obtain nationally representative data on risk behaviors among Korean adolescents. The present study used open-access public data available through the KYRBS website, and thus was granted an exemption from ethical approval by Southwestern University of Finance and Economics (Approval number/Date: SWUFE 6/2025).

**Figure 1:**
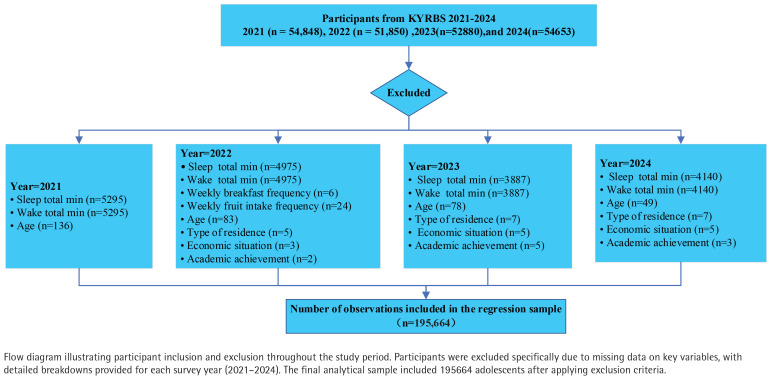
Flowchart of participants selection and exclusion, South Korea (2021–2024)

The sampling design stratifies schools into 117 strata based on 39 regional clusters and three school types (middle schools, general high schools, and specialized high schools). Within each stratum, students are proportionally distributed across grade levels in 800 participating schools. The analytic sample included respondents from the 2021 (n=54848), 2022 (n=51850), 2023 (n=52880), and 2024 (n=54653) survey waves, which were combined to construct a pooled cross-sectional dataset. After listwise deletion of missing data, 195664 participants remained for the primary analysis of sleep outcomes (Figure 1).

### Outcome variable

*Sleep duration*

According to the 2016 consensus statement of the American Academy of Sleep Medicine, the recommended sleep duration is 9–12 hours per day for children aged 6–12 years and 8–10 hours per day for adolescents aged 13–18 years^23^. In this study, the dependent variable was sleeping duration.

Sleep duration was derived from participants’ self-reported bedtimes and wake-up times on both weekdays and weekends, collected through the self-administered KYRBS questionnaire. Each reported time consisted of two components: hour (0–23) and minute (0–59). To standardize these variables, both bedtime and wake-up times were converted into a continuous measure expressed as the number of minutes elapsed since midnight. This was calculated as (hour × 60) + minutes (e.g. 23:10 = 23 × 60 + 10 = 1390 minutes).

Because sleep frequently extends past midnight, cases in which the wake-up time was earlier than the bedtime (e.g. bedtime at 23:00 and wake-up at 06:30) were corrected by adding 1440 minutes (24 hours) to the wake-up time. Total sleep duration in minutes was then computed as the difference between wake-up and bedtime and subsequently converted into decimal hours by dividing by 60. This procedure was applied separately to weekday and weekend data.

To capture overall sleep patterns, a weighted weekly average was constructed to reflect the proportion of school days and weekends, using the formula:


$$\frac{\left(5\;\times \text{ weekday sleep duration}\right)\;+\;\left(2\;\times \text{ weekend sleep duration}\right)}{7}$$

### Independent variables

*Weekly average days of SHS exposure*

SHS exposure was the independent variable in this study. SHS exposure was quantified based on the number of days participants were exposed. Specifically, responses to the following questionnaire items were used: ‘In the last 7 days, how many days have you inhaled the smoke of someone else’s cigarette in your home?’ and ‘In the last 7 days, how many days have you inhaled someone else’s cigarette smoke indoors (shops, restaurants, malls, concert halls, PC rooms, karaoke bars, etc.) that are not at home or school?’. Responses to these items were originally coded from 1 to 8, corresponding to 0 to 7 days of exposure. Each item was recorded by subtracting 1, so that the original coding of 1–8 corresponded to 0–7 days. This process yielded two variables: the number of days of SHS exposure at home in the past week and the number of days of SHS exposure in other indoor locations in the past week. An additional variable representing cumulative SHS exposure days was then calculated by summing these two variables. The cumulative exposure variable was created by summing reported exposure days across home and other indoor locations, resulting in a measure ranging from 0 to 14 days that reflects total exposure frequency across multiple environments.

### Moderator variable

*Tobacco use*

In this study, the variable ‘tobacco use’ was operationalized using a composite measurement approach: initial screening was conducted through three dichotomous variables (yes/no) to assess respondents’ lifetime use of ordinary cigarettes, nicotine-containing liquid e-cigarettes, and heated tobacco products; these responses were then integrated into a single polytomous variable ranging from 0 to 3, where 0 indicates no use of any tobacco product and 3 indicates use of all three types.

### Controlled variables

The selection of control variables was informed by a review of the extant literature on adolescent health and sleep^3^, as well as biological plausibility regarding potential confounding factors. A set of controlled variables was included at the individual, school, and family levels. Individual-level characteristics included sex (male=1, female=0), age, academic performance, and health-related behaviors: breakfast frequency, fruit intake frequency, and total physical activity. Academic achievement was self-rated by participants using a 5-point Likert scale ranging from 1 (poor) to 5 (excellent). Academic performance was reverse-coded on a 1–5 scale, with higher scores indicating better performance.

Participants reported the number of days they consumed breakfast in the past seven days, excluding days when only milk or juice was consumed. Meals including bread, meal replacement drinks, misstart (grain powder drink), porridge, or cereal were counted as breakfast. Responses ranged from 0 (no days) to 7 (every day). Higher values indicate more frequent breakfast consumption.

Fruit intake frequency was measured using the question: ‘In the past 7 days, how often did you consume fruit (excluding fruit juice)?’. Participants selected from seven options: 0 = none in the past 7 days, 1 = 1–2 times per week, 2 = 3–4 times per week, 3 = 5–6 times per week, 4 = once per day, 5 = twice per day, and 6 = three or more times per day.

Physical activity was assessed as the number of days participants engaged in at least 60 minutes of moderate-to-vigorous physical activity per day (PA_TOT) in the past seven days. Participants were asked: ‘In the past 7 days, regardless of the type of activity, on how many days did you engage in physical activity totaling at least 60 minutes that increased your heart rate or made you breathe harder?’. Responses formed a continuous variable ranging from 0 to 7. These were included because: 1) sex and age differences are known to affect both SHS exposure patterns and sleep requirements; 2) academic performance may reflect lifestyle patterns that influence both tobacco exposure opportunities and sleep habits; and 3) health behaviors tend to cluster and may confound the observed SHS-sleep relationship.

School-level control variables included grade, school type (girls’ school, co-educational, boys’ school), and school division (middle or high school). School division because educational environments significantly influence both social exposure contexts and sleep schedules through varying academic demands, institutional routines, and peer influences.

Family-level control variables included perceived economic status (1–5), with higher values indicating better economic conditions, and living arrangements, categorized as living with family, living with relatives, boarding or independent rental (including shared housing with friends), dormitory, or child welfare institution (orphanage, social welfare facility, or childcare center). These factors are well-established determinants of both household smoking behaviors and sleep environment quality.

### Statistical analysis

All analyses were performed using Stata 17.0 and included 195664 participants. First, descriptive analyses were conducted to summarize the characteristics of all variables, with categorical variables presented as frequencies and percentages, and continuous variables as means with standard deviations. Initial group comparisons between smokers and non-smokers were performed using t-tests for continuous variables and chi-squared tests for categorical variables.

The prevalence of tobacco use was calculated stratified by year, and box plots were generated to visualize the relationship between sleep duration and age. Second, since average sleep duration is a continuous variable, this study employed ordinary least squares (OLS) regression models for analysis. All statistical tests were two-tailed and the significance levels were denoted as follows: *p<0.1, **p<0.05, ***p<0.01. Specifically, control variables were sequentially incorporated at the individual, school, and household levels to analyze the association between SHS exposure and average sleep duration. Sleep duration was categorized into average daily sleep duration during the week, average daily sleep duration on weekends, and average daily sleep duration on weekdays. Models 1–3, respectively, represent the relationship between the frequency of SHS exposure and these three types of sleep duration. All control variables, along with time and location fixed effects, were included in Models 1–3.

Additionally, to complement the primary analyses and assess the robustness of the findings, we conducted supplementary logistic regression analyses using a dichotomized sleep duration variable. Sleep duration was dichotomized at the sample median, where values below the median were classified as ‘shorter sleep’ and values at or above the median as ‘longer sleep’. These models, which adjusted for the same comprehensive set of control variables and fixed effects as the primary OLS models, examined the association between SHS exposure and the odds of having shorter sleep duration. The results are reported as odds ratios (ORs) with 95% confidence intervals. Finally, an interaction term between SHS exposure and tobacco use was introduced to examine the moderating role of tobacco use in the relationship between SHS exposure and sleep duration.

## RESULTS

### Descriptive statistical analysis

After excluding 18567 participants with incomplete data on key variables, the analytical sample comprised 195664 Korean adolescents for the final analysis. Table 1 provides descriptive statistics for the key variables. The overall sample consisted of 50.9% males and 49.1% females, indicating a relatively balanced gender distribution. The average age of participants was approximately 15.0 years. Most adolescents reported their family’s economic status as middle-income (47.2%), followed by high-income (30.4%) and low-income (9.4%). Academic achievement was self-rated on a 5-point scale, with the largest proportion of students perceiving themselves as ‘average’ (29.9%), followed by ‘below average’ (22.5%) and ‘good’ (25.5%). The majority of students attended co-educational schools (54.9%), while girls’ schools and boys’ schools accounted for 29.2% and 15.9%, respectively. Most students resided with family members, and were enrolled in middle school rather than high school. All variables showed significant differences between groups (p<0.001), indicating a clear contrast in characteristics between smokers and non-smokers. Prominent features of the smoking group included shorter sleep duration, higher SHS exposure, poorer academic performance, and lower frequency of breakfast and fruit intake per week.

**Table 1:** Descriptive statistics for all variables, by smoking or not, South Korea, 2021–2024 (N=195664)

*Variables*	*Overall (N=195664)*			*No smoking (N=177063)*	*Smoking (N=18601)*	*Mean test*	
	*Proportion*	*n*	*Mean*	*Mean*	*Mean*	*Diff*	*p*
**Sleep duration** (hours)							
Weekly average			6.902	6.938	6.558	0.380	<0.01
Weekday average			6.243	6.289	5.808	0.481	<0.01
Weekend average			8.548	8.560	8.431	0.129	<0.01
**Weekly average days of secondhand smoke exposure** (0–14)			1.954	1.852	2.925	-1.073	<0.01
**Gender**			0.509	0.492	0.671	-0.179	<0.01
Male	0.509	96046					
Female	0.491	99618					
**Age** (years) (range 12–18)			15.035	14.943	15.913	-0.970	<0.01
**Academic achievement** (range 1–5)			3.109	3.154	2.678	0.476	<0.01
Poor	0.091	17813					
Below average	0.225	43987					
Average	0.299	58524					
Good	0.255	49835					
Excellent	0.130	25505					
**Weekly breakfast frequency (days)**			4.663	4.733	3.998	0.735	<0.01
**Weekly fruit intake frequency**			2.989	3.019	2.704	0.315	<0.01
0	0.113	22196					
1–2 times per week	0.327	63971					
3–4 times per week	0.284	55485					
5–6 times per week	0.104	20411					
Once per day	0.102	19956					
Twice per day	0.044	8670					
Three or more times per day	0.025	4975					
**Weekly physical activity frequency** (days)			3.176	3.138	3.541	-0.403	<0.01
**Grade** (range 1–6)			3.351	3.261	4.212	-0.952	<0.01
The first grade of junior high school	0.181	35496					
The second grade of junior high school	0.181	35420					
The third grade of junior high school	0.178	34801					
The first grade of high school	0.164	32123					
The second grade of high school	0.156	30487					
The third grade of high school	0.140	27337					
**School type**			1.867	1.859	1.941	-0.082	<0.01
Girls’ school	0.292	57153					
Co-educational	0.549	107343					
Boys school	0.159	31168					
**Educational stage**			1.478	1.464	1.607	-0.142	<0.01
Middle school	0.522	102138					
High school	0.478	93526					
**Living status**			1.125	1.114	1.224	-0.110	<0.01
With family members	0.955	186815					
At relatives’ homes	0.005	899					
Boarding or renting independently	0.005	936					
In dormitory	0.033	6440					
In children’s welfare institution	0.003	574					
**Economic situation** (range 1–5)			3.398	3.411	3.271	0.140	<0.01
Economically disadvantaged family	0.018	3581					
Low-income family	0.094	18396					
Middle-income family	0.472	92278					
High-income family	0.304	59436					
Affluent family	0.112	21973					

Group differences were calculated using t-tests for continuous variables and chi-squared tests for categorical variable. Weekly physical activity frequency was measured as the number of days per week with at least 60 minutes of moderate-to-vigorous physical activity, with possible values ranging from 0 to 7 days.

* p<0.1,

** p<0.05,

*** p<0.01.

**Figure 2:**
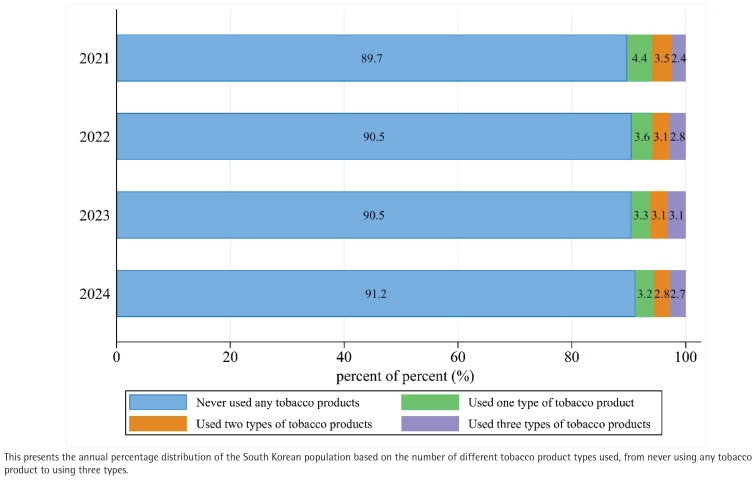
Trends in multi-tobacco product use, South Korea (2021–2024)

This Figure 2 illustrates the distribution of tobacco product usage patterns in South Korea from 2021 to 2024, based on a large-scale survey. The analysis revealed changes in tobacco use patterns from 2021 to 2024, with the proportion of adolescents never using tobacco products increasing from 89.7% to 91.2%, while use of one or two tobacco products decreased from 7.9% to 6.1%. The trend for use of three types of tobacco products appeared more variable. A slight increase was observed from 2021 (2.4%) to a peak in 2023 (3.1%), followed by a decrease to 2.7% in 2024.

As shown in Supplementary file Figure 1, SHS exposure among Korean adolescents showed significant fluctuations from 2021 to 2024, with mean exposure days increasing from 1.90 in 2021 to 2.18 in 2022, then declining to 2.04 in 2023 and further to 1.72 in 2024 (p for trend <0.001), representing an overall 9.4% decrease from the 2021 baseline.

**Figure 3:**
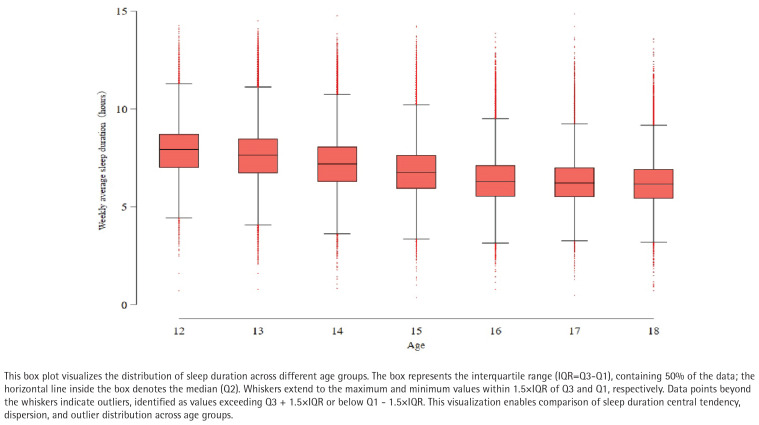
Weekly average sleep duration by age

An analysis of the box plots depicting adolescents’ weekly average, weekday average, and weekend average sleep duration revealed patterns associated with age (Figure 3). Overall, sleep duration showed a steady declining trend in median levels as age increased. This trend was most pronounced on weekdays, where sleep duration distributions across age groups were relatively concentrated, with fewer outliers, indicating that adolescents’ sleep schedules on weekdays are influenced by external constraints (such as school start times), resulting in relatively smaller individual differences. In contrast, sleep duration on weekends exhibited greater variability, specifically reflected in wider interquartile ranges and more outliers, often toward longer sleep durations (Supplementary file Figures 2 and 3). This suggests that adolescents have the opportunity to compensate for weekday sleep deprivation by extending their sleep on weekends, but this compensatory behavior varies significantly among individuals, particularly in older groups (e.g. 17–18 years), where some individuals’ weekend sleep duration far exceeds that of their peers. Taken together, the findings indicate that as adolescents age, their overall sleep duration decreases, while the disparity between their sleep patterns on weekdays and weekends also widens, reflecting the complex interaction between societal schedules and sleep needs during adolescence.

**Figure 4:**
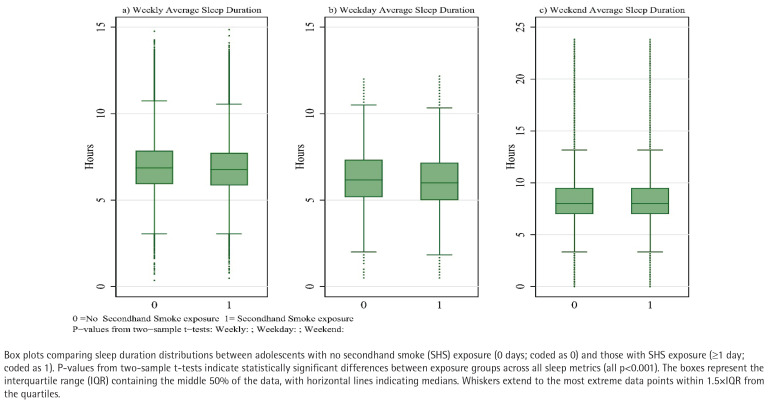
Sleep duration by SHS exposure status among Korean adolescents, 2021-2024

Figure 4 presents the distribution of sleep duration stratified by SHS exposure status, revealing consistent and significant differences between exposure groups across all sleep metrics. Adolescents with SHS exposure (≥1 day) demonstrated substantially shorter sleep duration compared to their non-exposed counterparts (0 days). Visual inspection of the box plots demonstrates a clear downward shift in the distribution of sleep duration among exposed adolescents, with lower medians and quartile values evident in all three panels. Formal statistical comparisons confirmed these visual patterns, with two-sample t-tests indicating significantly shorter sleep duration in the SHS exposure group (all p<0.001). The reduction in sleep duration associated with SHS exposure was most pronounced for weekday sleep (Figure 4b), followed by weekly average sleep (Figure 4a), while the difference, though still significant, was somewhat attenuated for weekend sleep (Figure 4c).

The consistency of these patterns across different sleep metrics reinforces the robustness of the association between SHS exposure and reduced sleep duration identified in our regression analyses.

### Regression results

Table 2 examines the relationship between SHS exposure and sleep duration. Model 1 indicates that SHS exposure has a significant association with weekly average sleep duration, with a regression coefficient of -0.032. After sequentially adding control variables in Models 2 through 4, the results remain significant at the 95% confidence level. All control variables are included in Model 4. The findings suggest that, holding other conditions constant, each one-unit increase in SHS exposure is associated with an average reduction of 0.027 hours in adolescents’ sleep duration (p<0.01).

**Table 2:** Ordinary least squares regression results on the association between secondhand smoke exposure and weekly average sleep duration among adolescents, South Korea, 2021–2024 (N=199564)

*Variables*	*Weekly average sleep duration*			
	*Model 1* *β (95% CI)*	*Model 2* *β (95% CI)*	*Model 3* *β (95% CI)*	*Model 4* *β (95% CI)*
**Secondhand smoke exposure**	-0.032***(-0.034 – -0.030)	-0.026***(-0.028 – -0.024)	-0.026***(-0.028 – -0.024)	-0.027***(-0.029 – -0.024)
**Gender**		0.404***(0.391–0.416)	0.417***(0.404–0.430)	0.418***(0.404–0.431)
**Age** (years)		-0.316***(-0.319 – -0.312)	-0.075***(-0.088 – -0.062)	-0.076***(-0.088 – -0.063)
**Academic achievement**		-0.041***(-0.047 – -0.036)	-0.048***(-0.053 – -0.042)	-0.044***(-0.050 – -0.039)
**Weekly breakfast frequency**		0.037***(0.035–0.040)	0.037***(0.035–0.040)	0.037***(0.035–0.040)
**Weekly fruit intake frequency**		0.002 (-0.002–0.007)	0.002(-0.003–0.006)	0.003(-0.002–0.007)
**Weekly physical activity frequency**		0.011***(0.008–0.014)	0.008***(0.005–0.011)	0.008***(0.005–0.011)
**Grade**			-0.249***(-0.263 – -0.236)	-0.250***(-0.264 – -0.237)
**School type**			-0.031***(-0.041 – -0.020)	-0.030***(-0.041 – -0.020)
**Educational stage**			-0.077***(-0.091 – -0.064)	-0.078***(-0.091 – -0.064)
**Living status**				0.017***(0.007–0.027)
**Economic situation**				-0.019***(-0.026 – -0.012)
**Year FE**	Yes	Yes	Yes	Yes
**City FE**	Yes	Yes	Yes	Yes
**Total**, n	195664	195664	195664	195664
**adjusted R^2^**	0.0086	0.1698	0.1766	0.1768

The adjusted R^2^ values reported reflect the variance in sleep duration explained by the full model. These values are consistent with behavioral epidemiology, where single models typically explain limited portions of variance in complex health outcomes, after adjusting for the number of predictors. Our focus was on estimating the specific effect of secondhand smoke exposure within a robust statistical framework. Coefficients β represent the change in sleep duration (hours) associated with a one-unit change in each predictor variable. For categorical variables, reference categories are specified in the Methods section. For categorical variables, the reference category is indicated as follows: female (for gender), economically disadvantaged family (for economic status), and Girls’ school (for school type). All other categories are interpreted relative to these reference groups. Year FE: year fixed effects. City FE: city fixed effects.

* p<0.1,

** p<0.05,

*** p<0.01.

Table 3 presents the analysis of the relationship between SHS exposure frequency and different sleep duration metrics after adjusting for potential confounding factors. Findings from Models 1 to 3 indicate a significant negative association (p<0.01) between SHS exposure and sleep duration when other variables are held constant. Specifically, each one-day increase in SHS exposure is associated with significant reductions in sleep duration: weekly average sleep decreases by 0.027 hours (β= -0.027; 95% CI: -0.029 – -0.024, p<0.01), weekday sleep decreases by 0.030 hours (β= -0.030; 95% CI: -0.032 – -0.028, p<0.01), and weekend sleep decreases by 0.018 hours (β= -0.018; 95% CI: 0.023 – -0.013, p<0.01).

**Table 3:** Ordinary least squares regression results on the association between secondhand smoke exposure and sleep duration among adolescents, South Korea, 2021–2024 (N=199564)

*Variables*	*Weekly average sleep duration*	*Weekday average sleep duration*	*Weekend average sleep duration*
	*Model 1* *β (95% CI)*	*Model 2* *β (95% CI)*	*Model 3* *β (95% CI)*
**Secondhand smoke exposure**	-0.027***(-0.029 – -0.024)	-0.030***(-0.032 – -0.028)	-0.018***(-0.023 – -0.013)
**Gender**	0.418***(0.404–0.431)	0.508***(0.496–0.520)	0.192***(0.164–0.219)
**Age** (years)	-0.076***(-0.088 – -0.063)	-0.069***(-0.081 – -0.057)	-0.091***(-0.118 – -0.064)
**Academic achievement**	-0.044***(-0.050 – -0.039)	-0.018***(-0.023 – -0.013)	-0.110***(-0.121 – -0.098)
**Weekly breakfast frequency**	0.037***(0.035–0.040)	0.049***(0.047–0.051)	0.009***(0.004–0.014)
**Weekly fruit intake frequency**	0.003 (-0.002–0.007)	0.007***(0.002–0.011)	-0.007 (-0.016–0.002)
**Weekly physical activity frequency**	0.008***(0.005–0.011)	0.010***(0.007–0.013)	0.005 (-0.001–0.011)
**Grade**	-0.250***(-0.264 – -0.237)	-0.273***(-0.286 – -0.261	-0.192***(-0.220 – -0.164)
**School type**	-0.030***(-0.041– -0.020)	-0.035***(-0.045 – -0.025)	-0.019*(-0.040–0.003)
**Educational stage**	-0.078***(-0.091 – -0.064)	-0.092***(-0.104 – -0.079)	-0.043***(-0.071 – -0.015)
**Living status**	0.017***(0.007–0.027)	0.021***(0.012–0.031)	0.006 (-0.016–0.027)
**Economic situation**	-0.019***(-0.026 – -0.012)	-0.013***(-0.019 – -0.006)	-0.034***(-0.050 – -0.019)
**Year FE**	Yes	Yes	Yes
**City FE**	Yes	Yes	Yes
**Total**, n	195664	195664	195664
**Adjusted R^2^**	0.1768	0.2294	0.0308

The adjusted R^2^ values reported reflect the variance in sleep duration explained by the full model. These values are consistent with behavioral epidemiology, where single models typically explain limited portions of variance in complex health outcomes, after adjusting for the number of predictors. Our focus was on estimating the specific effect of secondhand smoke exposure within a robust statistical framework. Coefficients β represent the change in sleep duration (hours) associated with a one-unit change in each predictor variable. For categorical variables, reference categories are specified in the Methods section. For categorical variables, the reference category is indicated as follows: female (for gender), economically disadvantaged family (for economic status), and Girls’ school (for school type). All other categories are interpreted relative to these reference groups. Year FE: year fixed effects. City FE: city fixed effects.

* p<0.1,

** p<0.05,

*** p<0.01.

Beyond SHS exposure, several control variables demonstrated significant associations with sleep duration. Significant gender differences were observed, with males showing longer sleep duration across all metrics, particularly on weekdays (β=0.508; 95% CI: 0.496–0.520, p<0.01). Age exhibited a consistent negative association with sleep duration (β= -0.076, 95% CI: -0.088 – -0.063, p<0.01 for weekly average; β= -0.091; 95% CI: -0.118 – -0.064, p<0.01 for weekend). Academic achievement showed varying associations across different sleep metrics, with significant negative effects on weekly average (β= -0.044; 95% CI: -0.050 – -0.039, p<0.01) and weekend sleep duration (β= -0.110; 95% CI: 0.121 – -0.098, p<0.01, but a weaker effect on weekday sleep (β= -0.018; 95% CI: 0.023 – -0.013, p<0.01). Among health behaviors, breakfast frequency showed strong positive associations, particularly for weekday sleep (β=0.049; 95% CI: 0.047–0.051, p<0.01). School-level factors revealed that higher grade levels were strongly associated with reduced sleep duration, especially on weekdays (β= -0.273; 95% CI: 0.286 – -0.261, p<0.01). These findings highlight the importance of controlling for these confounders when examining the relationship between SHS exposure and sleep outcomes.

After adjusting for all control variables and fixed effects, SHS exposure remained significantly associated with increased odds of shorter sleep duration across all three sleep metrics. Each additional day of SHS exposure was associated with 3.4% higher odds of having shorter weekly average sleep duration (AOR=1.034; 95% CI: 1.031–1.038), 4.0% higher odds for shorter weekday sleep duration (AOR=1.040; 95% CI: 1.036–1.044), and 1.5% higher odds for shorter weekend sleep duration (AOR=1.015; 95% CI: 1.011–1.018). Several variables demonstrated consistent patterns with the primary analyses. Female adolescents showed significantly lower odds of shorter sleep duration across all models (weekly: AOR=0.568; weekday: AOR=0.503; weekend: AOR=0.853). Age was positively associated with shorter sleep duration, with older adolescents having approximately 10.5%, 9.0%, and 5.3% higher odds for weekly, weekday, and weekend shorter sleep duration, respectively (Table 4).

**Table 4:** Logit regression results on the relationship between the secondhand smoke exposure and sleep duration (above vs below median) among adolescents, South Korea, 2021–2024 (N=195664)

*Variables*	*Weekly average sleep duration*	*Weekday average sleep duration*	*Weekend average sleep duration*
	*Model 1* *AOR (95% CI)*	*Model 2* *AOR (95% CI)*	*Model 3* *AOR (95% CI)*
**Secondhand smoke exposure**	1.034***(1.031–1.038)	1.040***(1.036–1.044)	1.015***(1.011–1.018)
**Gender**	0.568***(0.556–0.580)	0.503***(0.492–0.514)	0.853***(0.836–0.871)
**Age** (years)	1.105***(1.082–1.128)	1.090***(1.068–1.113)	1.053***(1.033–1.074)
**Academic achievement**	1.062***(1.052–1.071)	1.034***(1.025–1.044)	1.079***(1.070–1.088)
**Weekly breakfast frequency**	0.950***(0.946–0.953)	0.935***(0.932–0.939)	0.996**(0.993–1.000)
**Weekly fruit intake frequency**	1.005 (0.998–1.012)	0.998 (0.991–1.005)	1.022***(1.015–1.029)
**Weekly physical activity frequency**	0.976***(0.971–0.981)	0.975***(0.971–0.980)	0.996*(0.991–1.000)
**Grade**	1.429***(1.399–1.460)	1.507***(1.474–1.540)	1.174***(1.150–1.199)
**School type**	1.066***(1.048–1.084)	1.063***(1.045–1.081)	1.021***(1.005–1.038)
**Educational stage**	1.124***(1.100–1.148)	1.132***(1.107–1.156)	1.050***(1.029–1.072)
**Living status**	0.980**(0.965–0.997)	0.980**(0.963–0.996)	1.027***(1.011–1.043)
**Economic situation**	1.035***(1.023–1.047)	1.036***(1.024–1.049)	1.054***(1.042–1.066)
**Year FE**	Yes	Yes	Yes
**City FE**	Yes	Yes	Yes
**Total, n**	195664	195664	195664
**pseudo R^2^**	0.1168	0.1389	0.0259

AOR: adjusted odds ratio. Year FE: year fixed effects. City FE: city fixed effects.

* p<0.1,

** p<0.05,

*** p<0.01.

### The analysis of moderating effects

Table 5 incorporates tobacco use as a moderating variable in the analysis. The results demonstrate that, after controlling for other variables, SHS exposure maintains a significant direct negative association with sleep duration. Meanwhile, tobacco use as a moderating variable also shows a strong independent negative association with both weekday and weekly average sleep duration. Analysis of the main effects indicates that each additional day of SHS exposure was associated with significant reductions in sleep duration across all measures: weekly average sleep duration decreased by 0.027 hours (β= -0.027; 95% CI: -0.030 – -0.025, p<0.01), weekday sleep duration decreased by 0.029 hours (β= -0.029, 95% CI: -0.031– -0.027, p<0.01), and weekend sleep duration decreased by 0.023 hours (β= -0.023, 95% CI: -0.028 – -0.018, p<0.01).

**Table 5:** Moderating effect of tobacco use on the association between secondhand smoke exposure and sleep duration, South Korea, 2021–2024 (N=195664)

*Variables*	*Weekly average sleep duration*	*Weekday average sleep duration*	*Weekend average sleep duration*
	*Model 1* *β (95% CI)*	*Model 2* *β (95% CI)*	*Model 3* *β (95% CI)*
**Secondhand smoke exposure**	-0.027***(-0.030 – -0.025)	-0.029***(-0.031 – -0.027)	-0.023***(-0.028 – -0.018)
**Tobacco use**	-0.076***(-0.089 – -0.063)	-0.109***(-0.122 – -0.097)	0.008 (-0.020–0.035)
**Secondhand smoke exposure × tobacco use**	0.007***(0.004–0.010)	0.005***(0.003–0.008)	0.011***(0.005–0.016)
**Gender**	0.424***(0.411–0.43)	0.519***(0.507–0.532)	0.186***(0.158–0.214)
**Age** (years)	-0.075***(-0.088 – -0.062)	-0.068***(-0.080 – -0.056)	-0.092***(-0.118 – -0.065)
**Academic achievement**	-0.047***(-0.052 – -0.041)	-0.022***(-0.027 – -0.017)	-0.108***(-0.120 – -0.096)
**Weekly breakfast frequency**	0.037***(0.034–0.039)	0.048***(0.046–0.050)	0.009***(0.004–0.014)
**Weekly fruit intake frequency**	0.002 (-0.002–0.006)	0.005***(0.001–0.009)	-0.006 (-0.015–0.003)
**Weekly physical activity frequency**	0.009***(0.006–0.012)	0.011***(0.008–0.014)	0.004 (-0.002–0.011)
**Grade**	-0.248***(-0.261 – -0.234)	-0.270***(-0.282 – -0.257)	-0.194***(-0.222 – -0.166)
**School type**	-0.030***(-0.041 – -0.020)	-0.035***(-0.045 – -0.025)	-0.019*(-0.040–0.003)
**Educational stage**	-0.077***(-0.091 – -0.064)	-0.091***(-0.103 – -0.078)	-0.043***(-0.071 – -0.015)
**Living status**	0.018***(0.008–0.029)	0.024***(0.015–0.034)	0.003 (-0.018–0.025)
**Economic situation**	-0.018***(-0.026 – -0.011)	-0.012***(-0.019 – -0.005)	-0.035***(-0.051 – -0.020)
**Year FE**	Yes	Yes	Yes
**City FE**	Yes	Yes	Yes
**Total**, n	195664	195664	195664
**Adjusted R^2^**	0.1773	0.2309	0.0309

This table examines the moderating role of tobacco use through the inclusion of an interaction term (secondhand smoke exposure × tobacco use). The positive and statistically significant interaction coefficients indicate that tobacco use moderates the relationship between secondhand smoke exposure and sleep duration. The adjusted R^2^ values reported reflect the variance in sleep duration explained by the full model, after adjusting for the number of predictors. These values are consistent with behavioral epidemiology, where single models typically explain limited portions of variance in complex health outcomes. Our focus was on estimating the specific effect of secondhand smoke exposure within a robust statistical framework. Coefficients β represent the change in sleep duration (hours) associated with a one-unit change in each predictor variable. For categorical variables, reference categories are specified in the Methods section. For categorical variables, the reference category is indicated as follows: female (for gender), economically disadvantaged family (for economic status), and Girls’ school (for school type). All other categories are interpreted relative to these reference groups. Year FE: year fixed effects. City FE: city fixed effects.

* p<0.1,

** p<0.05,

*** p<0.01.

The interaction term (SHS exposure × tobacco use) shows significant positive coefficients across all three sleep duration models. Notably, the interaction terms between SHS exposure and tobacco use were consistently positive and significant across all models: weekly average (β=0.007; 95% CI: 0.004–0.010, p<0.01), weekday (β=0.005; 95% CI: 0.003–0.008, p<0.01), and weekend (β=0.011; 95% CI: 0.005– 0.016, p<0.01). This finding indicates that tobacco use significantly moderates the strength of the association between SHS exposure and sleep duration. Whether an individual uses tobacco products serves as an important boundary condition determining the extent to which their sleep health is affected by SHS exposure. For tobacco users, the additional negative impact of SHS exposure is relatively smaller, potentially due to developed tolerance to nicotine and other substances, or because their sleep quality has already been affected by their own smoking behavior.

## DISCUSSION

Analyzing large-scale data from the KYRBS from 2021 to 2024, this study examined the relationship between SHS exposure and sleep duration in Korean adolescents. The research specifically focused on how adolescents’ tobacco use behavior may regulate this association. The findings revealed a clear and negative association between SHS exposure and reduced sleep duration among adolescents, which was consistent across various sleep metrics (weekly, weekday, weekend). Notably, adolescents’ own tobacco use was found to significantly moderate the impact of SHS on sleep duration, offering novel empirical insights into the individual mechanisms underlying the effects of SHS on sleep quality.

First, according to the descriptive statistical analysis, all variables showed significant differences between groups, indicating clear contrasts in multiple characteristics between smokers and non-smokers. The smoking group exhibited features such as shorter sleep duration, higher SHS exposure, poorer academic performance, and lower weekly frequencies of breakfast and fruit intake, which is consistent with previous studies^11,22,24^. Meanwhile, trend analysis of tobacco use revealed a yearly increase in the proportion of adolescents who had never used any tobacco products, along with a general decline in the use of one or two types of tobacco products. Although the use of three tobacco product types showed greater fluctuation, the overall trend indicates a decline in tobacco use among Korean adolescents. Furthermore, analysis of sleep patterns showed that adolescents generally compensate for weekday sleep deprivation by extending sleep on weekends, but this compensatory behavior varies significantly among individuals, particularly among older adolescents aged 17–18 years, some of whom had substantially longer weekend sleep duration than their peers. In summary, as adolescents grow older, their total sleep duration shows a declining trend.

Furthermore, this study confirmed a direct association between exposure to SHS and sleep duration among adolescents, revealing that with each incremental rise in SHS exposure, there were notable reductions in both weekly and weekday sleep duration. These findings suggests that even after controlling for other potential influencing factors, lower environmental exposure to SHS is associated with better adolescent sleep health. This observation aligns with prior research indicating that constituents of SHS such as nicotine, carbon monoxide, and suspended particulate matter can disrupt sleep patterns by stimulating the respiratory system and inducing hypoxemia^3,16,19,25^. Adolescent smoking behavior commonly exhibits substantial social clustering^26,27^, with adolescents frequently encountering firsthand and SHS in environments where peers smoke. This dual exposure may intensify adverse impacts on sleep quality by worsening disruptions in the sleep-wake cycle due to a synergistic effect of nicotine. Notably, the impact on weekday sleep appears to be more pronounced, possibly due to the combination of academic pressures and routine^28,29^. Conversely, although weekend sleep was less affected, the influence remained significant, underscoring the persistent nature of SHS exposure beyond weekdays^30,31^. Therefore, efforts to improve adolescent sleep health should not only focus on active smoking cessation, but also emphasize minimizing SHS exposure in both household and public settings, particularly through the creation of smoke-free sleeping environments.

Subsequent analysis revealed that tobacco use significantly influenced the association between exposure to SHS and sleep duration among adolescents. Specifically, a positive interaction coefficient suggests that the adverse impact of SHS on sleep duration is less pronounced in adolescents who use tobacco. This finding, though seemingly paradoxical, could be attributed to the development of nicotine tolerance and adaptive adjustments in the sleep–wake cycle^32^. Essentially, habitual smokers may have adapted their sleep patterns over time due to prolonged exposure to neuroactive substances like nicotine, potentially diminishing their sensitivity to similar elements in SHS^33^. However, sleep health risks may not be lower in this group; the combination of active smoking and SHS exposure could impose more intricate physiological burdens. Nevertheless, in this study, there is no simple additive effect on sleep duration. Conversely, smoking adolescents may frequently experience sleep disturbances due to active smoking, such as difficulties falling asleep or fragmented sleep^11^. Moreover, their baseline sleep duration is typically low^16^, suggesting that the impact of SHS exposure may be relatively minimal. Based on this finding, it is reasonable to consider that the sleep health implications of SHS exposure may differ between adolescent tobacco users and non-users. For tobacco users, whose sleep patterns are already influenced by direct nicotine intake, the relative impact of additional SHS exposure may be less pronounced. In contrast, for non-users, who lack such physiological adaptation, SHS exposure may play a more direct and significant role in sleep duration reduction.

### Limitations

This study has limitations. First, the data were obtained from self-reported questionnaires, potentially introducing social expectation bias or recall bias. Second, the cross-sectional nature of the design precludes the determination of causal direction. Although it is plausible that SHS affects sleep quality, reverse causation remains possible – for instance, sleep-deprived adolescents may be more likely to seek out tobacco-related stimuli or encounter environments with SHS. Additionally, the study population was drawn from a single national context, which may limit the generalizability of the findings to other countries or cultural settings. Future studies would benefit from longitudinal designs, along with objective measures such as wrist actigraphy for sleep assessment and air monitors for quantifying SHS exposure, to strengthen causal inference and measurement accuracy.

## CONCLUSIONS

This study, based on a sizable nationally representative sample, provides evidence for a significant negative association between exposure to SHS and sleep duration in Korean adolescents. It also clarifies that individual tobacco use behavior plays a moderating role in this association. These results enhance comprehension of the intricate relationships among various tobacco exposure routes and sleep quality.

## Supplementary Material



## Data Availability

The data supporting this research are available from the following sources: https://www.kdca.go.kr/yhs/, http://yhs.cdc.go.kr.
