# Supplementary material for: Secondhand smoke exposure and sleep disturbances among Korean adolescents: A nationally representative cross-sectional study
Source: Tob Induc Dis. 2025 Dec 31;23:10.18332/tid/213717. doi: 10.18332/tid/213717 (PMC12754694; doi:10.18332/tid/213717)
Supplement: Supplementary file 1 [file TID-23-200-s1.pdf]

Supplementary file

Supplementary Figure 1. Secondhand Smoke Exposure Trends in Korean Adolescents, (2021-2024) (N=195664)

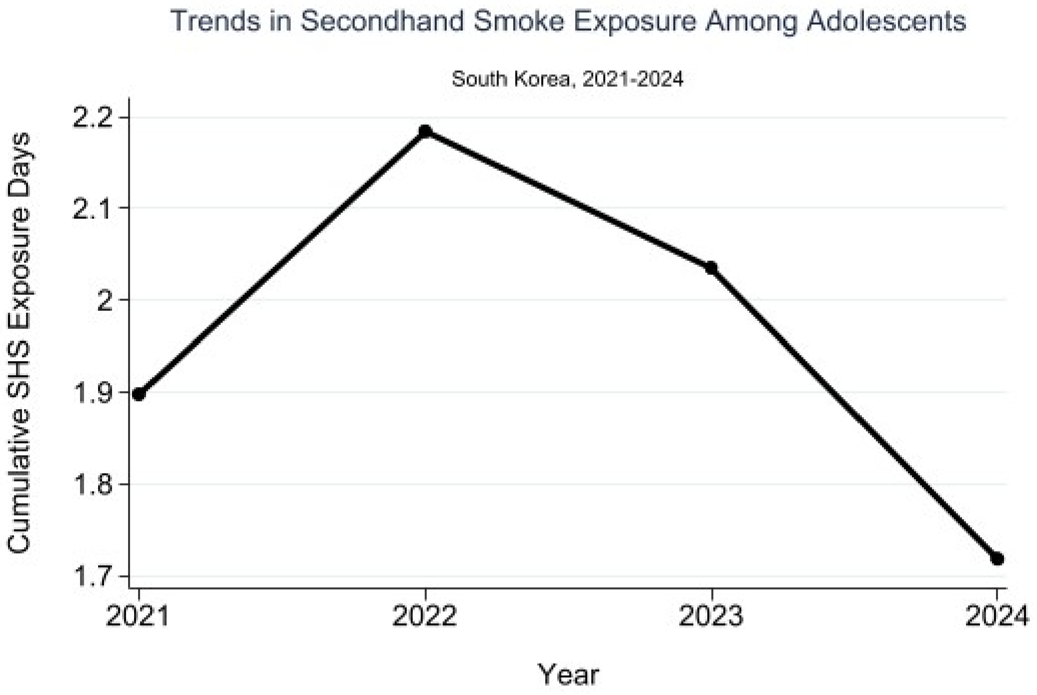

Supplementary Figure 2. Weekday average sleep duration

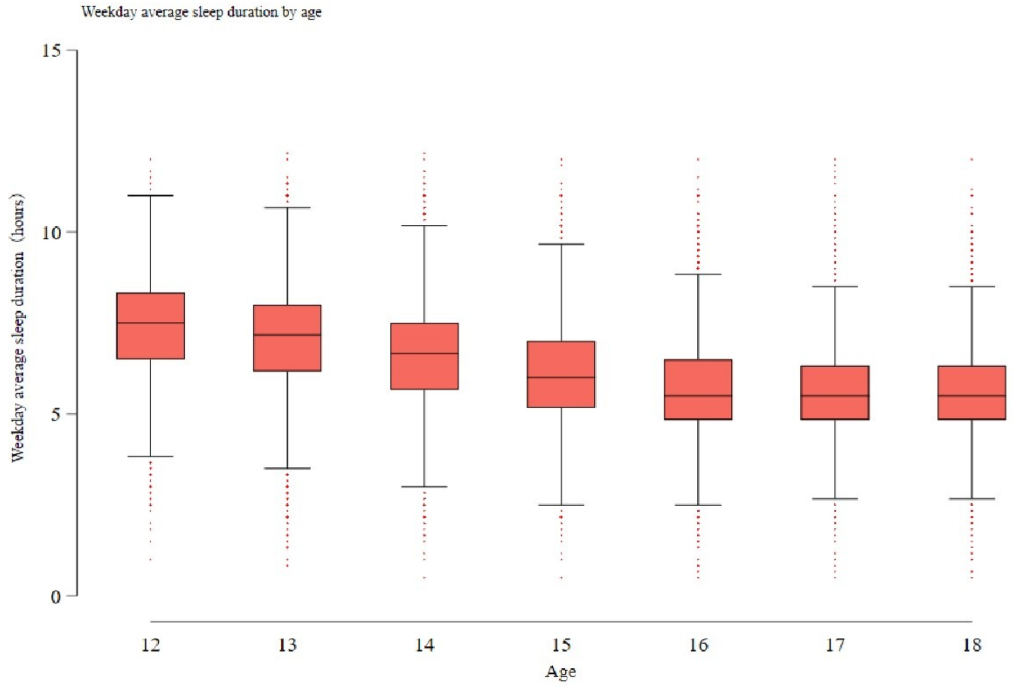

Supplementary Figure 3. Weekend average sleep duration

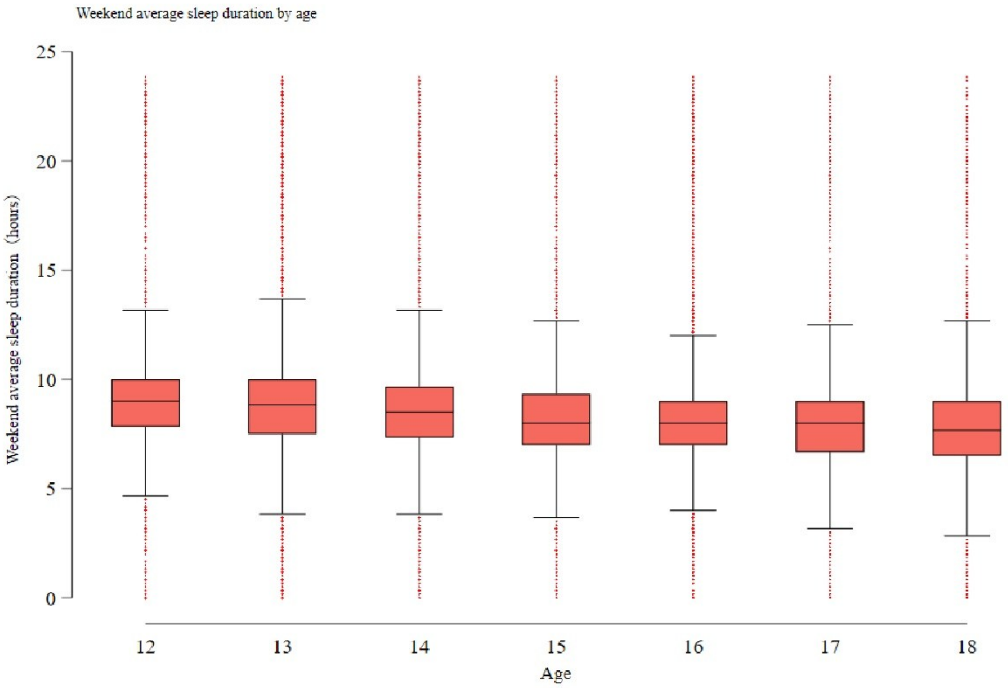

© 2025 Hua F. et al.
